# En bloc aorto-iliac vessel resection and bifurcated graft replacement for localized lymph node recurrence after rectal cancer surgery

**DOI:** 10.1016/j.jvscit.2025.102018

**Published:** 2025-10-15

**Authors:** Hironari Shibahara, Jun Yokote, Masato Yamakawa, Shinichi Ashida, Ryo Yamamoto, Yuya Urano

**Affiliations:** a Department of Cardiovascular Surgery, Ogaki Municipal Hospital, Ogaki, Japan; b Department of Surgery, Ogaki Municipal Hospital, Ogaki, Japan; c Department of Tumor Pathology, Nagoya University Graduate School of Medicine, Nagoya, Japan; d Department of Pathology, Ogaki Municipal Hospital, Ogaki, Japan

**Keywords:** Bifurcated graft replacement, En bloc resection, Localized lymph node recurrence, Rectal cancer

## Abstract

Major vessel resection and reconstruction for locally advanced recurrent colorectal cancer can achieve favorable outcomes when performed in specialized centers. A 67-year-old female initially underwent laparoscopic low anterior resection for rectal cancer (T4N1M0). Two years postoperatively, localized lymph node recurrence involving the left common iliac vessels was diagnosed. En bloc resection of the involved iliac vessels and bifurcated graft replacement was performed. At 30 months of follow-up, no recurrence was observed. En bloc aorto-iliac vessel resection with bifurcated graft replacement can be a feasible and effective treatment strategy for selected cases of localized lymph node recurrence after rectal cancer surgery.

According to the Japanese Society for Cancer of the Colon and Rectum Guidelines 2019,^1^ localized lymph node recurrence should be considered for surgery only when the disease is otherwise controlled, and the surgical indication should be determined after careful consideration of both the operative risk and the postoperative quality of life.

Reports from specialized centers have shown that major vessel resection and reconstruction for locally advanced recurrent colorectal cancer, as well as pelvic exenteration for malignancy, can achieve favorable outcomes and extend survival.^2^^,^^3^ However, the most appropriate technique for vascular reconstruction after vessel resection remains undefined.

We present a case of localized lymph node recurrence involving the common iliac vessels after rectal cancer surgery, successfully treated with en bloc tumor resection and vascular reconstruction, resulting in favorable clinical outcomes.

## Case report

**Fig 1:**
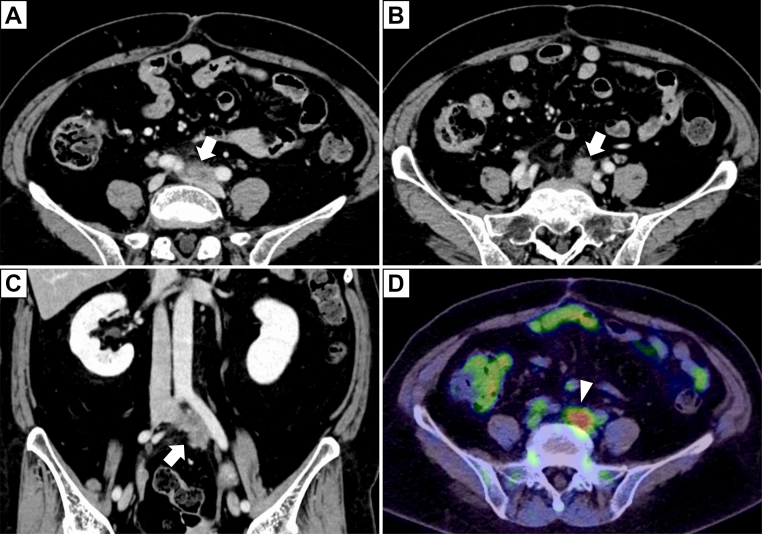
Preoperative image. Preoperative contrast-enhanced computed tomography (CT) and positron emission tomography-CT of the abdomen. **(A** and **B)** On the axial CT slice, enlargement of the left common iliac lymph node was noted (*white arrow*), with infiltration extending from the terminal aorta to the bifurcation of the left common iliac artery (CIA). **(C)** On the coronal CT slice, infiltration of the left common iliac lymph node into the left common iliac vein (CIV) was observed (*white arrow*). **(D)** Positron emission tomography-CT revealed accumulations in the enlarged common iliac lymph node (*white arrowhead*).

A 67-year-old female with a history of type 2 diabetes mellitus, who had undergone laparoscopic low anterior resection for rectal cancer (T4N1M0) 2 years earlier, presented with an elevated serum CA 19-9 level of 71.8 U/mL after her initial surgery, without any symptoms, such as left lower extremity swelling. Contrast-enhanced computed tomography (CT) revealed an enlarged left common iliac lymph node invading the common iliac vessels (Fig 1, *A*-*C*). Positron emission tomography-CT revealed abnormal fluorodeoxyglucose uptake at the same lesion (Fig 1, *D*), with no evidence of distant metastasis. These findings supported the diagnosis of localized lymph node recurrence involving the left common iliac vessels. Given the lesion’s localized nature and its indolent progression over 2 years, en bloc resection of the tumor and affected vessels was planned, with vascular reconstruction using a prosthetic graft, and without adjuvant chemotherapy or radiotherapy.

**Fig 2:**
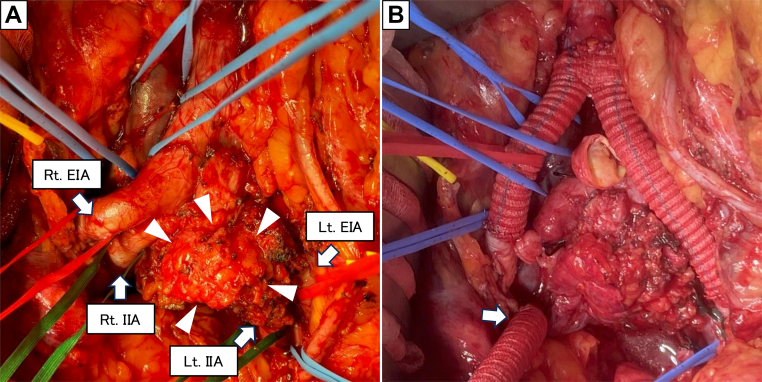
Intraoperative findings. **(A)** After retroperitoneal incision, a recurrent tumor was identified in the left common iliac lymph node (*white arrowhead*), with infiltration observed around the left common iliac (CIA), external iliac (*EIA*), and internal iliac arteries (*IIA*) and veins. **(B)** Reconstruction was performed using a synthetic vascular graft, extending peripherally to both bilateral EIAs. The right IIA was deemed reconstructable, and an 8-mm synthetic graft was end-to-end anastomosed to the right IIA for graft interposition (*white arrow*).

**Fig 3:**
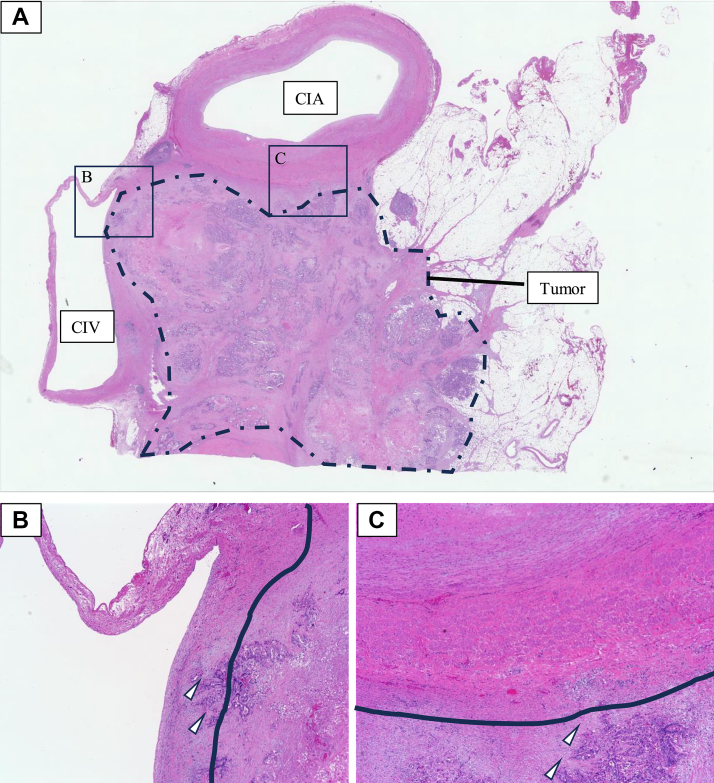
Histopathological findings of the resected specimen. **(A)** Adenocarcinoma cells had infiltrated near the common iliac artery and vein (*CIA, CIV*) (hematoxylin and eosin staining). **(B)** Adenocarcinoma cells (*white arrowheads*) were observed infiltrating the CIV (The *solid line* represents the contour of the vein wall). Tumor thrombus or bland thrombus was not identified in the lumen. **(C)** Adenocarcinoma cells (*white arrowheads*) were observed near the CIA (The *solid line* represents the contour of the arterial wall).

A midline laparotomy was performed, and the tumor was found to extend from the left common iliac artery (CIA) and common iliac vein (CIV) to the left internal and external iliac vein (IIV/EIV). The bilateral external iliac arteries (EIAs), internal iliac arteries (IIAs), the inferior vena cava, the bilateral CIVs and ureters were encircled with vessel loops (Fig 2, *A*). After ligating the left IIA, a bifurcated graft (J Graft 16 × 8 mm; Japan Lifeline, Shinagawa) was placed from the abdominal aorta to the bilateral EIAs to maintain perfusion to both lower limbs, with additional graft interposition to the right IIA (Fig 2, *B*). The left IIV, EIV, and CIV were ligated, and the tumor was carefully dissected away from the anterior vertebral body. En bloc resection was then carried out, including the left CIA and CIV, extending to the left EIA, IIA, EIV, and IIV. Macroscopically, the recurrent tumor spared the aortic bifurcation, right CIA, EIA, and IIA. Graft interposition was completed by anastomosing the graft limb to the right IIA. Histopathological examination confirmed lymph node metastasis of adenocarcinoma, with infiltration of adenocarcinoma cells surrounding the CIA and CIV (Fig 3). Macroscopically and on histological examination, no tumor thrombus or bland thrombus was identified within the venous lumen.

**Fig 4:**
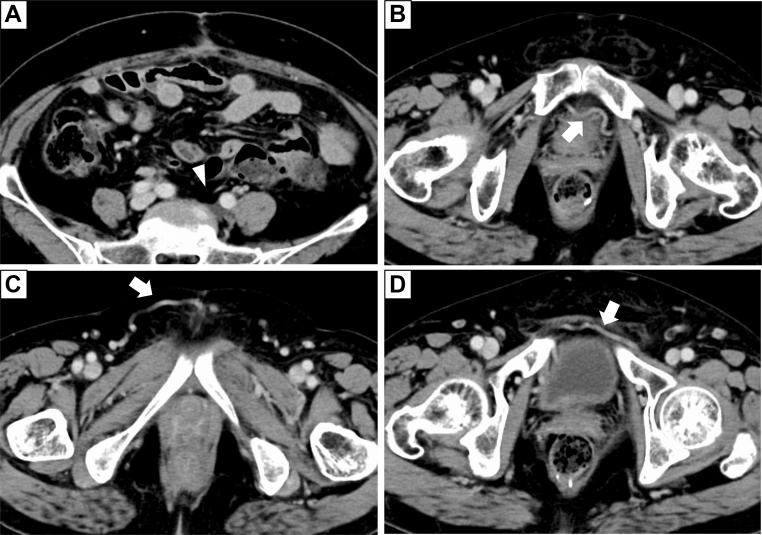
Postoperative contrast-enhanced computed tomography (CT) at 3 months after surgery. **(A)** Left common iliac vein (CIV) and left common iliac lymph node were completely resected, and there was no evidence of recurrence (*white arrowhead*). **(B)** Development of collateral veins from left to right within the vesical venous plexus was observed (*white arrow*). **(C)** Development of collateral veins from the superficial external pudendal vein was observed (*white arrow*). **(D)** Development of the pubic vein was observed (*white arrow*).

The patient was discharged on postoperative day 12 with mild left leg edema, which resolved after the use of compression stockings. A follow-up CT at 3 months demonstrated the formation of collateral venous pathways from the left to the right side, involving the vesical venous plexus, superficial external pudendal vein, and pubic vein (Fig 4). At 30 months postoperatively, no evidence of recurrence was observed, and the patient remained free of leg edema.

## Discussion

Surgical resection and reconstruction of major vessels for recurrent or locally advanced primary cancer have been shown to achieve favorable outcomes and extended survival in specialized centers. Abdelsattar et al reported that, among 406 consecutive patients undergoing surgery for locally recurrent colorectal cancer, 12 patients underwent resection for locally advanced disease involving the aortoiliac axis, including the IIA, CIA, EIA, abdominal aorta, IIV, and EIV.^2^ They demonstrated that R0 resection was achieved in over 50% of patients, 30-day mortality was nil, and overall and disease-free survival at 4 years was 55% and 45%.^2^ Similarly, Brown et al reported that, among 336 patients undergoing pelvic exenteration, 21 patients (6.3%) underwent en bloc vascular excision of 29 vessels for tumor involvement.^3^ They reported median overall and disease-free survival times of 34 and 26 months, respectively, and concluded that advanced pelvic tumors involving iliac vessels should not be considered contraindications to curative surgery in specialized institutions.

Techniques for arterial reconstruction in iliac lesions include bifurcated graft replacement, synthetic interposition grafting, femoral-femoral bypass, and primary anastomosis.^2^, ^3^, ^4^, ^5^ Abdelsattar et al also reported that seven patients required arterial reconstruction (three aorta, five CIA, and three EIA).^2^ Hashimoto et al described a case of paraaortic lymph node metastasis 4 years after colon cancer surgery that was treated with en bloc resection and graft replacement of the abdominal aorta.^4^ In our case, because preoperative CT demonstrated the recurrent tumor extended from the left CIA to the EIA, IIA, EIV, and IIV, bifurcated graft replacement was performed to ensure tumor-free margins. Furthermore, because there was no risk of intraoperative contamination from bowel resection, a synthetic graft was selected for arterial reconstruction.

Venous reconstruction after en bloc vessel resection remains controversial.^5^, ^6^, ^7^, ^8^, ^9^ An understanding of venous collateral anatomy and drainage routes is crucial to prevent lower limb complications when major veins are removed. Umeoka et al examined iliac vein obstruction caused by deep vein thrombosis, direct invasion by pelvic malignancy, or lymphadenopathy, and demonstrated various collateral pathways through analysis of CT and magnetic resonance imaging.^6^ They noted that even in cases of unilateral occlusion of the CIV or simultaneous occlusion of the EIV and IIV, the external pudendal vein or the pubic vein might provide communication between the bilateral iliac channels. Leg edema remains a concern in cases where venous reconstruction is not performed. Adelani et al compared outcomes in 14 patients with lower extremity soft tissue sarcomas invading major vessels who underwent limb-preserving tumor resection with or without venous reconstruction.^7^ They found no significant difference in the incidence of leg edema between the two groups. Additionally, several case reports suggested that venous reconstruction after en bloc resection may not be essential.^5^^,^^8^^,^^9^ In our case, leg edema was temporary, and follow-up CT demonstrated the development of collateral veins from the left to the right side. Thus, even when en bloc resections without venous reconstruction—including iliac veins—are performed, collateral pathways from the pelvic venous plexuses can develop to prevent significant venous congestion.

Overall, this case highlights that the patient remained recurrence-free for 30 months postoperatively with a favorable clinical course, and that postoperative CT evaluation of venous return after venous resection proved valuable, suggesting that venous reconstruction may not always be necessary. If complete resection is not feasible, systemic therapy, chemoradiotherapy, or radiotherapy should be considered as treatment options.^1^ In contrast, if complete resection is achievable, en bloc vessel resection combined with arterial reconstruction may be considered a viable treatment approach.

## Conclusions

En bloc aorto-iliac vessel resection with bifurcated graft replacement may serve as an effective surgical strategy for localized lymph node recurrence after rectal cancer surgery, provided that cases are carefully selected.

## Declaration of generative AI and AI-assisted technologies in the writing process

During the preparation of this work, the authors used ChatGPT (OpenAI) to improve the readability and language of the manuscript. After using this tool, the authors reviewed and edited the content as needed and take full responsibility for the content of the published article.

## Funding

None.

## Disclosures

None.
